# Bovine Holo-Beta-Lactoglobulin Cross-Protects Against Pollen Allergies in an Innate Manner in BALB/c Mice: Potential Model for the Farm Effect

**DOI:** 10.3389/fimmu.2021.611474

**Published:** 2021-03-05

**Authors:** Sheriene Moussa Afify, Isabella Pali-Schöll, Karin Hufnagl, Gerlinde Hofstetter, Maha Abdel-Rafea El-Bassuoni, Franziska Roth-Walter, Erika Jensen-Jarolim

**Affiliations:** ^1^ The Interuniversity Messerli Research Institute of the University of Veterinary Medicine Vienna, Medical University Vienna and University Vienna, Vienna, Austria; ^2^ Laboratory Medicine and Immunology Department, Faculty of Medicine, Menoufia University, Shibin El Kom, Egypt; ^3^ Institute of Pathophysiology and Allergy Research, Center of Pathophysiology, Infectiology and Immunology, Medical University of Vienna, Vienna, Austria; ^4^ Biomedical International R+D GmbH, Vienna, Austria

**Keywords:** allergy, beta-lactoglobulin, holo-BLG, ligands, tolerance, cross-protection, immune resilience, protective farm effect

## Abstract

The lipocalin beta-lactoglobulin (BLG) is a major protein compound in cow’s milk, and we detected it in cattle stable dust. BLG may be a novel player in the farm protective effect against atopic sensitization and hayfever. In previous studies, we demonstrated that only the ligand-filled holo-form of BLG prevented sensitization to itself. Here, we investigated whether holo-BLG could, in an innate manner, also protect against allergic sensitization to unrelated birch pollen allergens using a murine model. BALB/c mice were nasally pretreated four times in biweekly intervals with holo-BLG containing quercetin–iron complexes as ligands, with empty apo-BLG, or were sham-treated. Subsequently, mice were intraperitoneally sensitized two times with apo-BLG or with the unrelated birch pollen allergen apo-Bet v 1, adjuvanted with aluminum hydroxide. After subsequent systemic challenge with BLG or Bet v 1, body temperature drop was monitored by anaphylaxis imaging. Specific antibodies in serum and cytokines of BLG- and Bet v 1-stimulated splenocytes were analyzed by ELISA. Additionally, human peripheral blood mononuclear cells of pollen allergic subjects were stimulated with apo- versus holo-BLG before assessment by FACS. Prophylactic treatment with the holo-BLG resulted in protection against allergic sensitization and clinical reactivity also to Bet v 1 in an unspecific manner. Pretreatment with holo-BLG resulted in significantly lower BLG-as well as Bet v 1-specific antibodies and impaired antigen-presentation with significantly lower numbers of CD11c+MHCII+ cells expressing CD86. Pretreatment with holo-BLG also reduced the release of Th2-associated cytokines from Splenocytes in BLG-sensitized mice. Similarly, *in vitro* stimulation of PBMCs from birch pollen allergic subjects with holo-BLG resulted in a relative decrease of CD3+CD4+ and CD4+CRTh2 cells, but not of CD4+CD25+CD127− Treg cells, compared to apo-BLG stimulation. In conclusion, prophylactic treatment with holo-BLG protected against allergy in an antigen-specific and -unspecific manner by decreasing antigen presentation, specific antibody production and abrogating a Th2-response. Holo-BLG therefore promotes immune resilience against pollen allergens in an innate manner and may thereby contribute to the farm protective effect against atopic sensitization.

## Introduction

Cow’s milk allergy (CMA) is associated with a low quality of life in children and their families, as milk and milk products are considered essential food in early lifetime. As milk avoidance can be difficult (1), different preventive strategies to reduce the allergenicity of major allergens in cow’s milk have been conducted all over the world (2–9).

The phenomenon of CMA is in striking contrast to studies showing that consumption of unprocessed cow’s milk is considered to represent an important factor associated with the protective effect of cattle farms against atopic sensitization, asthma, and hayfever (10–13).

Milk processing, especially pasteurization, can affect the physiologic structure of several milk proteins, thereby increasing their allergenic potential (12, 14, 15). Heating milk above 65°C structurally alters the thermolabile milk proteins, in particular the whey fraction, and causes aggregates (16). This leads to an increase in the antigenicity of the whey protein beta-lactoglobulin (BLG) (16) and the appearance of several new epitopes on BLG (17) during protein unfolding (18). Several other milk constituents may be affected by processing, too. For example, during the defatting process involving centrifugation and homogenization, the milk lipid fraction, *e.g*. *ω*-3 polyunsaturated fatty acids, which is considered to be a precursor of anti-inflammatory mediators (19), is diminished.

BLG belongs to the lipocalin protein family (20, 21), which is capable of carrying molecules such as retinoids (22), fatty acids, hormones, vitamins, and iron-chelating agents (20, 23, 24) in their large, calyx-like pocket (25). In our previous studies, we showed that the holo-BLG loaded with the flavonoid quercetin–iron complex is not allergenic (22). Holo-BLG rather created a tolerogenic environment through promotion of regulatory cells (23) by delivering ligands, thereby activating the anti-inflammatory aryl hydrocarbon receptor (AHR) pathway and down-tuning the antigen presentation skills of antigen presenting cells. BLG is not only present in milk, but is also secreted in the cattles’ urine. Its presence can be detected in air samples and in dust samples in and around cattle stables (26) and (Pali-Scholl et al., manuscript in review).

Here, we went a step further, showing *in vivo* that the spiked holo-BLG is not an allergen, but protects against the onset of allergies in an antigen-specific as well as antigen-non-specific manner, similar to the observed allergy-protective farm effect.

## Materials and Methods

### Preparation of Apo-BLG

Commercially available bovine beta-lactoglobulin (≥90% pure, Sigma Aldrich, Steinheim, Germany) was dialyzed four times against 10 µM deferoxamine mesylate (DFO) by using snakeskin dialysis tube (ThermoScientific, MWCO 3.5 K), followed by four times dialyzation against deionized water.

### Generation of Holo-BLG

The holo-form of BLG was generated by incubating apo-BLG with flavonoid quercetin–iron complexes (FeQ2) in a molar ratio BLG:quercetin:iron of 1:2:1 as previously described (23).

### Animals

5–7 weeks old female BALB/c mice were purchased from Charles River (Sulzfeld, Germany), maintained on milk-free chow and treated under conventional housing conditions according to the European Community rules of animal care. All experiments were approved by the Animal Experimentation Ethics Committee of the University of Vienna and the Ministry of Education, Science and Culture (BMWF-66.009/0133-WF/V/3b/2016).

### Experimental Design: Intranasal Prophylaxis and Protection Against the Same Allergen (BLG)

Sample sizes for the mouse experiments were based on the literature. No randomization was performed and protocols were designed as follows:

Prophylaxis: Mice (n = 11 per group) were intranasally (i.n.) pretreated with 10 µl per mouse (5 µl per nostril) containing apo-BLG (10 µg of apo-BLG (0.5 nM) plus 0.3 mg of deferoxamine (0.5 nM) to prevent loading of BLG during nasal application) or holo-BLG, corresponding to BLG loaded with the flavonoid quercetin–iron complex (10 µg BLG plus 338 ng quercetin and 28 ng iron) four times on two consecutive days at 14 days interval or sham-pretreated with distilled water (n = 10).

**Figure 1:**
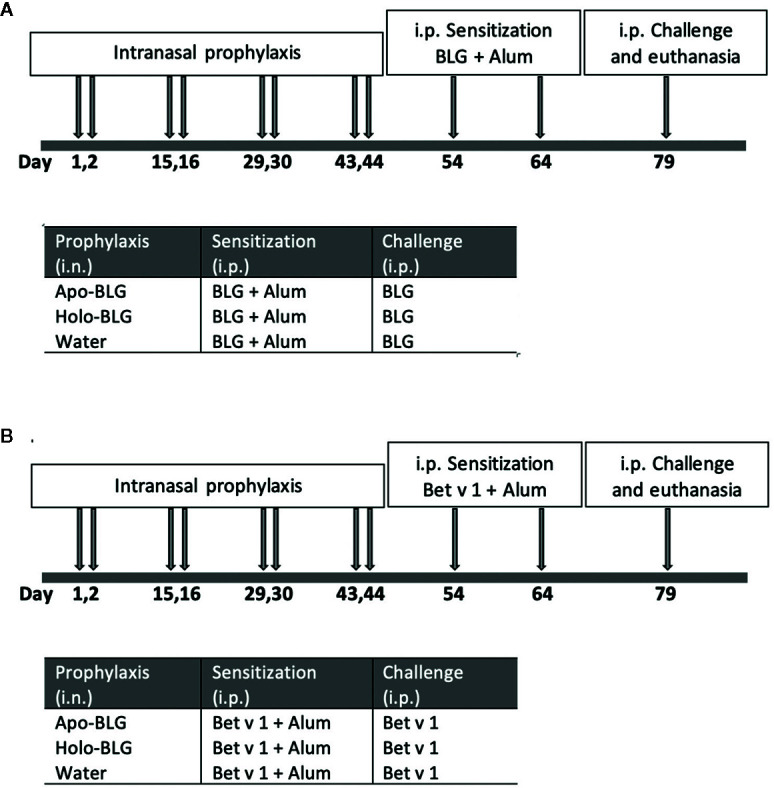
Schematic overview of the *in vivo* protocols. **(A)** Intranasal prophylactic treatment of mice to protect against sensitization to the same allergen (BLG). Mice were intranasally treated four times in biweekly intervals with either apo-BLG (n = 11), holo-BLG (n = 11), or water (as a control, n = 10). This was followed by two i.p.-sensitization steps with with BLG (5 μg/mouse) adjuvanted with 50 μl aluminum hydroxide within ten days. Thereafter, mice were i.p. challenged with apo-BLG (50 μg/mouse) and allergic response was monitored before euthanasia. **(B)** Intranasal prophylactic treatment of mice to protect against sensitization to an unrelated, non-milk allergen (Bet v 1). Mice were pre-treated as in protocol A, but were subsequently immunized twice i.p. with Bet v 1 (5 μg Bet v 1/mouse adjuvanted with 50 μl aluminum hydroxide). Thereafter, they were challenged with Bet v 1 (50 μg Bet v 1/mouse i.p.), and body temperature as well as physical activity was monitored before euthanasia.

Sensitization: For systemic sensitization, BLG (5 μg/mouse) adjuvanted with 50 μl aluminum hydroxide (alum, Serva, Heidelberg, Germany), was intraperitoneally (i.p.) injected two times in a 10-day interval. Two weeks after the last sensitization, all mice were intraperitoneally (i.p.) challenged with apo-BLG (50 μg/50 μl 0.9% NaCl/mouse) to induce an acute allergic response before they were sacrificed by gradual introduction of CO_2_. Pooled results from two independent experiments were compared. A schematic overview of the experimental design is depicted in **Figure 1A**.

### Experimental Design: Intranasal Prophylaxis and Protection Against Allergen Bet v 1

The experimental design of the second experiment is comparable to the one described above (**Figure 1B**). Shortly, a new/another set of mice were i.n. pretreated four times on two consecutive days at 14 days interval with distilled water as sham-treatment, apo-BLG (20 µg) or holo-BLG (20 µg BLG plus 676 ng quercetin and 56 ng iron). Each group consisted of 11 mice except for the sham-treated group, consisting of 10 mice. Two weeks after the last treatment, mice were i.p. sensitized with Bet v 1 (5 μg/mouse) adjuvanted with 50 μl aluminum hydroxide two times at 14-day intervals. Two weeks later, all mice were i.p. challenged with Bet v 1 (50 μg/50 μl 0.9% NaCl/mouse) to assess the occurrence of allergen-induced acute anaphylactic reaction. Results of two independently performed experiments were combined.

### *In Vivo* Evaluation of the Allergic Reaction: Anaphylaxis Read-Outs

Two weeks after the last sensitization, all mice were i.p. challenged with the allergen BLG or Bet v 1 (50 μg allergen/50 μl 0.9% NaCl). Over a period of 20 min, the anaphylactic shock-induced drop in body temperature and horizontal movement impairment were measured using a non-invasive heat imaging system (Biomedical Int. R+D, Vienna) (27). The severity of symptoms after the challenge was evaluated according to the scoring system previously described (16): 0 = no symptoms; 1 = scratching and rubbing around the nose and head; 2 = puffiness around the eyes and mouth, diarrhea, pilar erection, reduced activity and/or decreased activity with increased respiratory rate; 3 = wheezing, labored respiration and cyanosis around the mouth and the tail; 4 = No activity upon stimuli, tremor or convulsion; 5 = death. All assessments were performed in a blinded fashion.

### Antigen-Specific Antibodies

BLG and Bet v 1 specific IgG1, IgA, IgG2a, and IgE levels were measured by ELISA. Microtiter plates (Maxisorp, Nunc, Roskilde, Denmark) were coated (100 µl/well) with either BLG (10 µg/ml) or Bet v 1 (5 µg/ml) in carbonate coating buffer (pH 9.6) and incubated overnight at 4°C. Serum was added diluted 1:100 for IgG1, IgA, IgG2a, and 1:15 for IgE (100 µl/well) after washing and blocking with 1% BSA in PBS (RT/1 h) and incubated overnight at 4°C. Also, serial dilutions of mouse IgG1 (Southern Biotech, clone 15H6), IgG2a (Southern Biotech, clone HOPC-1), IgE (BD Biosciences, Clone IgE-3), and IgA (Southern Biotech, S107) standards were used and were directly coated. Monoclonal rat anti-mouse antibodies (eBiosciences), IgG1 (clone A85-1), IgG2a (clone R19-15), IgG2b (clone R12-3), IgA (clone c10-1), or IgE (clone R35-72) were applied, followed by incubation with polyclonal peroxidase-labeled goat anti-rat IgG antibodies (GE Healthcare). Tetramethylbenzidine (eBiosciences) was used as substrate and 1.8 M sulfuric acid was used as stop solution followed by optical density measurement at 450 nm.

### *In Vitro* Stimulation

After sacrifice, the spleens were harvested. Cell suspensions of individual spleens were prepared immediately by grinding and filtering through 40 μm nylon meshes (BD Biosciences, Schwechat, Austria) under sterile conditions. After erythrocytes lysis and washing, cells were counted and plated (4 × 10^6^ cells/well) in sterile round-bottom 48-well tissue culture plates (ThermoScientific) in RPMI medium. Splenocytes were stimulated with apo-BLG (5 and 25 μg/ml), Bet v 1 (25 μg/ml), and positive control concanavalin A (Con A) (2.5 μg/ml) or left unstimulated for 96 h at 37°C and 5% CO_2_. The supernatants were harvested and stored at −20°C until further use for cytokine measurement.

### Cytokines Detection

Cytokine concentrations in the undiluted supernatants of stimulated splenocytes were analyzed using an ELISA specific for murine IL-5, IL-10, IL-13, and IFN-**γ** (eBiosciences), according to the manufacturer’s instructions.

### Flow Cytometric Assessment of Co-Stimulatory Molecules on DCs

Single-cell suspensions of splenocytes (0.5 million cells) were incubated for 30 min under dim condition with anti-CD11c PE (eBioscience, clone N418), anti-MHC Class II I-Ad APC (clone AMS-32.1) and anti-CD86 FITC (clone GL1) in staining buffer (eBioscience). Afterwards, cells were washed two times with Hepes-buffer (20 mM Hepes, 150 mM NaCl, pH 7.2). Doublets were excluded, before gating on the living cells and using calcein-AM (Thermo-Fisher), as a living marker. Afterwards, cells were gated on CD11c+ in the living population, before gating on MHC Class II I-Ad+ CD86+ cells. Fluorescence Minus One (FMO) controls were used to identify gating boundaries. Acquisition and analysis were performed on a FACS Canto II flow cytometer (BD Bioscience, San Jose, CA, USA) using the FACSDiva Software 6.0.

### Isolation and Stimulation of Human PBMCs From Pollen Allergic Donors

The study was approved by the institutional ethics committee of the Medical University of Vienna and conducted in accordance with the Helsinki Declaration of 1975. Fourteen birch and/or grass pollen allergic volunteers donated 15 ml blood. All subjects gave their full written informed consent.

Heparin-treated blood was mixed with equal volumes of 0.9% sodium chloride solution before applying to 10 ml Ficoll-Paque (GE Healthcare) and centrifuged at 400 g for 30 min without brake as already described. After density gradient separation, the lymphocyte fraction was isolated and washed twice with 0.9% sodium chloride solution before being diluted to a concentration of 1 × 10^6^ cells/ml in DMEM medium containing neither phenol red nor fetal calf serum. Isolated PBMCs (0.5 Mio/ml) were incubated with apo-BLG (5 μM) and holo-BLG (5 μM BLG plus 10 μM quercetin and 5 μM iron) for 18 h.

Subsequently, cells were stained with combinations of Calcein Violet 450 AM (Thermo-Fisher) as a living marker, CD3-APC-Cy7 (Biolegend, clone SK7), CD4-PE-Cy7 (Biolegend, clone SK3), CD25-APC (biolegend, clone BC96), CD127-PE (Biolegend, clone A019D5) and CRTH2-FITC (Biolegend, clone BM16) and combinations of Calcein Violet 450 AM (Thermo-Fisher), CD14-APC (Biolegend, clone M5EZ), HLADR-PE (Biolegend, San Diego, Calif, clone L243PC), and CD86-PE-CY7 (Biolegend,clone IT2.2) for flow cytometric analysis. Doublets were excluded before gating the living lymphocytic population for CD3+ cells, and on the living monocytic population for CD14+ gating on the FSC/SSC plot. Samples were acquired by FACS Canto II machine (BD Bioscience, San Jose, CA, USA). Recorded events were analyzed with the FlowJo software version 10.3.

Supernatants of stimulated PBMCs were investigated for cytokines IL-2, IL-4, IL-5, IL-6, IL-10, IL-13, TNF-α; and IFN-**γ** by multiplex system in FACS (LEGENDplex™ Human Th1/Th2 Panel 8-plex, Biolegend).

### Statistical Analyses

Mouse groups and cellular studies were compared by performing ANOVA following the Tukey multiple comparisons test. Anaphylactic shock symptom score was analyzed with Kruskal–Wallis non-parametric test with Dunn’s multiple correction. To compare the effects of different treatments on primary cells, we applied repeated measures one-way ANOVA following the Tukey multiple comparisons test. All tests were two sided, and the results were considered significant when P was less than 0.05.

## Results

### Intranasal Application of Holo-BLG Decreases Sensitization Levels in Mice

**Figure 2:**
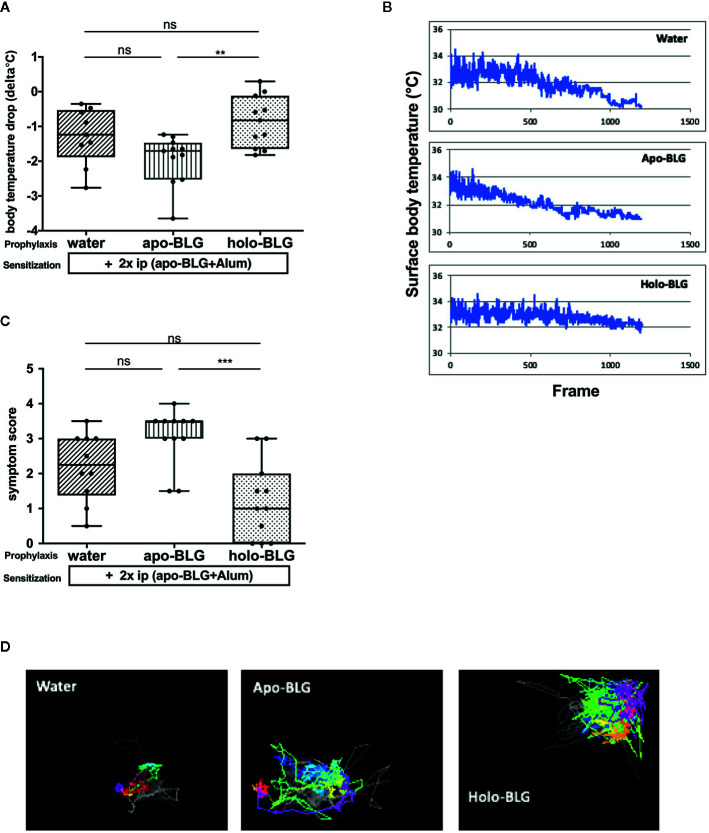
Holo-BLG pre-treatment protected against acute allergic symptoms to BLG. Pretreated mice were sensitized to BLG and thereafter challenged with BLG i.p., and allergic response was monitored. **(A)** Body temperature drop 20 min after i.p. challenge (ANOVA followed by Tukey’s multiple comparison test). **(B)** Representative examples of temperature drop during 20 min. observation period, x-axis represents number of frames (1 frame/s). **(C)** Anaphylactic shock symptom score (Kruskal–Wallis test). **(D)** Representative images of horizontal movements (lines) recorded by the imaging cage after systemic challenge with BLG in the differently treated groups. Pooled results from two independents experiments are shown. Panels in **(A, C)** show medians represented by a box whisker plot; **P < 0.01; ***P < 0.001; ns, non-significant.

We first investigated whether the loading condition of BLG is decisive for protection against BLG sensitization. Therefore, mice were intranasally treated four times at biweekly intervals with the ligand-filled holo-BLG, with empty apo-BLG, or sham-treated with water, then i.p. sensitized and challenged with BLG (scheme of treatment in **Figure 1**). As depicted in **Figures 2A, B**, treating mice with holo-BLG prior to BLG-sensitization decreased the sensitization level and therefore protected against clinical reactivity upon BLG challenge and significantly prevented the anaphylactic temperature drop when compared to the group pretreated with apo-BLG. This was also reflected by the significantly lower mean symptom score of one in the holo-BLG group compared to apo-BLG exposed group with a mean score of three (**Figure 2C**). Also, horizontal movements of individual mice monitored upon the specific allergen challenge showed that the physical capacity was better—though not reaching statistical significance—in mice exposed to holo-BLG compared to the group pretreated with apo-BLG in which the reduced horizontal physical activity reflected their anaphylactic reactions (**Figure 2D**).

### Holo-BLG Protects Against Sensitization to Itself With Strong Suppression of Th2 Cytokines

**Figure 3:**
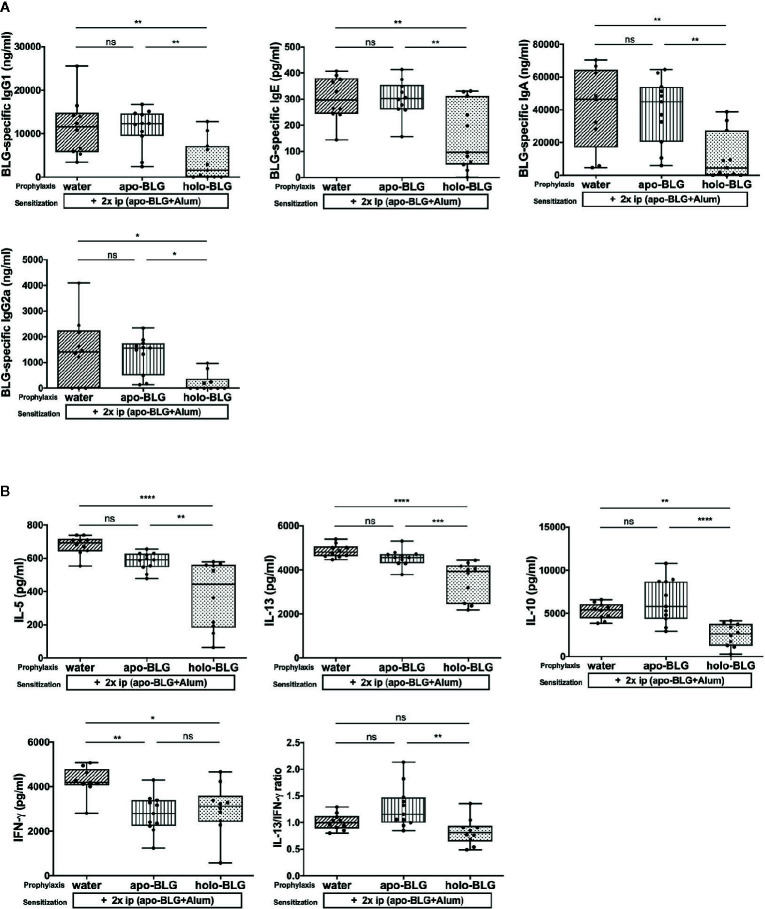
Holo-BLG pretreatment reduced antibody- and cytokine response in BLG-sensitized mice. **(A)** BLG-specific antibody-serum levels of mice treated with apo-BLG, holo-BLG, or water prior to BLG-sensitization and challenge with apo-BLG. **(B)** Concentrations of IL-5, IL-13, IL-10, IFN-*γ* and the ratio of IL-13/IFN-*γ* in supernatants of splenocytes stimulated with BLG for 4 days (37°C, 5% CO_2_). Pooled results from two independents experiments are shown. Groups were compared by ANOVA following Tukey’s multiple comparisons test. The panels show medians represented by a box whisker plot; *P < 0.05; **P < 0.01; ***P < 0.001; ****P < 0.0001; ns, non-significant.

We assessed which BLG-specific immunoglobulins were induced in the mice to understand the differences in the differently treated groups. Mice exposed to holo-BLG prior BLG-sensitization showed significantly lower levels of BLG-specific IgE, IgG1, IgA and IgG2a antibodies than the other groups despite the sensitization regimen applied (two i.p.-shots with BLG in combination with Alum as adjuvant) (**Figure 3A**).

In analogy to the humoral responses observed in the holo-BLG pretreated group, the BLG-stimulated splenocytes secreted significantly less IL-5, IL-13, but also IL-10 cytokines compared to apo-BLG (**Figure 3B**) due to targeted delivery of ligands to immune cells, synergizing in immune resilience (23). The Th2-associated cytokines, IL-5 and IL-13, were significantly lower in the holo-BLG groups, whereas IFN-**γ** levels were comparable between the apo- and holo-group. Hence, the Th2/Th1-ratio (IL-13/IFN-**γ**) was significantly higher in the apo-BLG group, emphasizing that the empty form of BLG evoked a strong Th2-response (**Figure 3B**).

### Impaired Antigen Presentation by Holo-BLG Pretreatment

**Figure 4:**
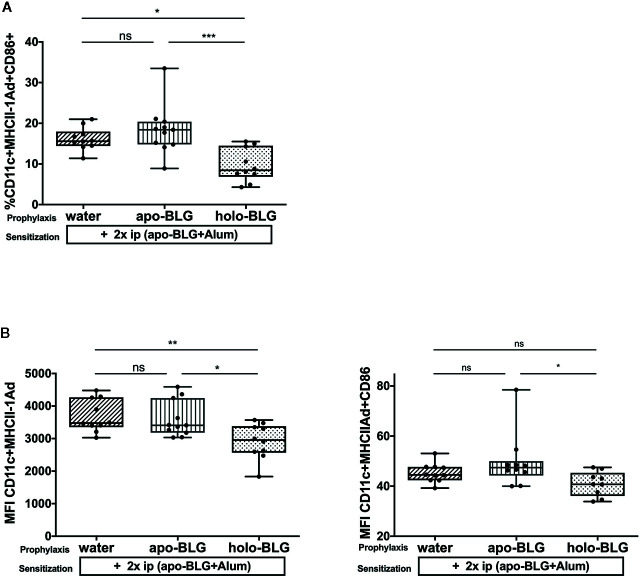
Impaired antigen presentation by holo-BLG pre-treatment in mice. **(A)** Splenocytes of the differently treated groups were analyzed for relative number of CD11c+ DCs expressing MHCII+CD86. **(B)** The mean fluorescence intensity (MFI) was measured for the co-stimulatory markers MHCII+CD86+ on CD11c+ DCs. Pooled results from two independent experiments are shown. Groups were compared by ANOVA after testing for normal distribution, followed by Tukey’s multiple comparisons test. The panels show medians and interquartile ranges represented by box whisker plots; *P < 0.05; **P < 0.01; ***P < 0.001; ns, non-significant.

Antigen-presenting cells are one of the first cells that encounter and process antigens and hence are critical for activating or suppressing the immune system. Consequently, we analyzed the co-stimulatory molecules on splenic dendritic cells (DCs) of each individual mouse. As depicted in **Figure 4**, the relative number of CD11+ dendritic cells expressing MHC Class II I-Ad+ and CD86+ was significantly reduced in mice pretreated with holo-BLG despite the strong subsequent sensitization scheme. Hence, holo-BLG pretreatment may impair the antigen presentation capacity of the dendritic cell population causing immune resilience (23) and may participate in tolerance induction, as antigen presentation in the absence of co-stimulatory molecules leads to anergy.

### Holo-BLG Cross-Protects Against Anaphylactic Reaction to Bet v 1

**Figure 5:**
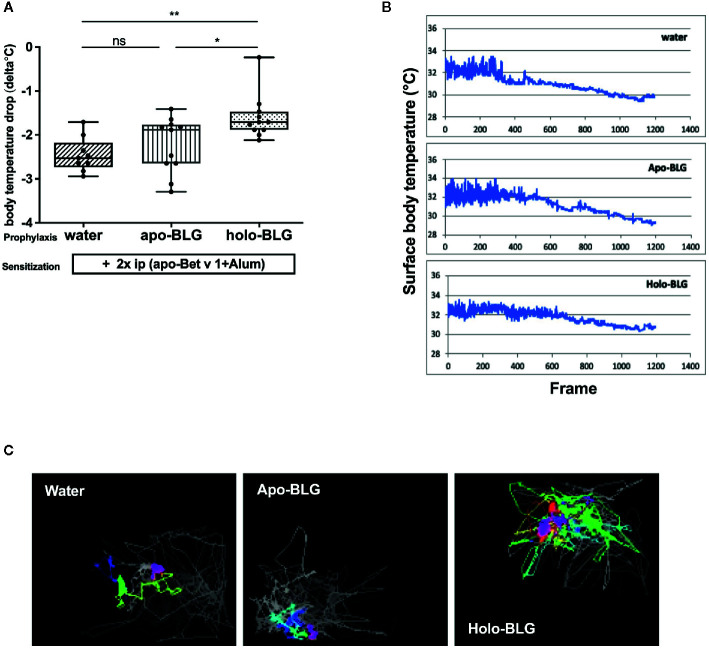
Holo-BLG treatment protected against acute allergic symptoms in an antigen-unspecific manner. Pretreated mice were sensitized to Betv1, thereafter challenged with Bet v 1, and the allergic response was monitored. **(A)** Body temperature drop determined 20 min after i.p. challenge (ANOVA followed by Tukey’s multiple comparison test). **(B)** Representative examples of temperature drop during the 20 min. observation period. **(C)** Representative images of horizontal movements (lines) recorded by the imaging cage after systemic challenge with Bet v 1 in the different treated groups, x-axis represents number of frames (1 frame/s). Combined results from two independents experimental rounds are shown. Medians with interquartile ranges are represented in box whisker plots; *P < 0.05; **P < 0.01; ns, non-significant.

As holo-BLG seemed to promote tolerogenic dendritic cells, we investigated whether this protective effect extends to the protection against other allergens. In a similar protocol as mentioned before, mice were either sham-treated with water, apo- or holo-BLG before sensitizing them twice, this time with the major birch pollen allergen Bet v 1 adjuvanted with Alum (**Figure 1B**). Indeed, as demonstrated in **Figures 5A, B**, initial mucosal exposure of mice to holo-BLG protected against clinical reactivity, preventing a body temperature drop (as a sign of anaphylaxis) compared to mice pretreated with apo-BLG or water alone before Bet v 1-sensitzation and challenge. This was also reflected in a significantly lower anaphylactic symptom score (**Supplementary Figure 1**) and protection from impaired physical activity (**Figure 5C**), compared to mice that had received apo-BLG or water prior to Bet v 1-sensitization.

### Holo-BLG Prevents Cross-Sensitization to Unrelated Allergen Bet v 1

**Figure 6:**
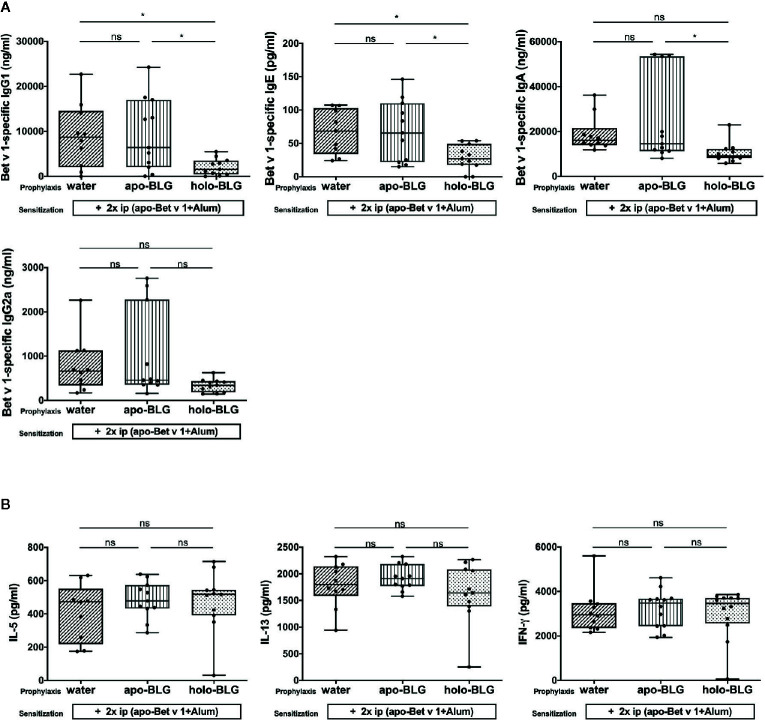
Holo-BLG pretreatment reduced antibody production, but not cytokine response in mice sensitized to Bet v1. **(A)** Bet v 1-specific antibody-levels were measured in serum of mice sensitized and challenged with Bet v 1 after being prophylactically treated intranasally with apo-BLG, holo-BLG, or water. **(B)** IL-5, IL-13, and IFN-*γ* concentrations were measured in supernatant after *ex vivo* stimulation of splenocytes with Bet v 1 for 4 days (37°C, 5% CO_2_). Pooled results from two independents experiments are shown. Groups were compared by ANOVA following Tukey’s multiple comparisons test. The panels show medians represented by a box whisker plot; *P < 0.05; ns, non-significant.

The prevention of allergic reactions upon pretreatment with holo-BLG before Bet v 1 sensitization was accompanied by reduced levels of Bet v 1-specific IgG1 and IgE, besides a trend towards lower Bet v 1-specific IgA, and IgG2a levels (**Figure 6A**). Hence, the reduced immune response to holo-BLG resulted in protection against allergic sensitization also to non-related antigens such as Bet v 1. However, we were not able to detect differences in the 96 h cytokine-secretion pattern in Bet v 1-stimulated splenocytes in the differently treated groups (**Figure 6B**). As in these late time points cytokines derive mostly from T-cells, the data suggest that prevention of allergy-development takes place rather during antigen presentation by impaired cross-presentation than on a T cellular level.

### Holo-BLG Hinders Antigen Presentation and Down-Regulates the Number of CD3+CD4+ Th2 Cells

**Figure 7:**
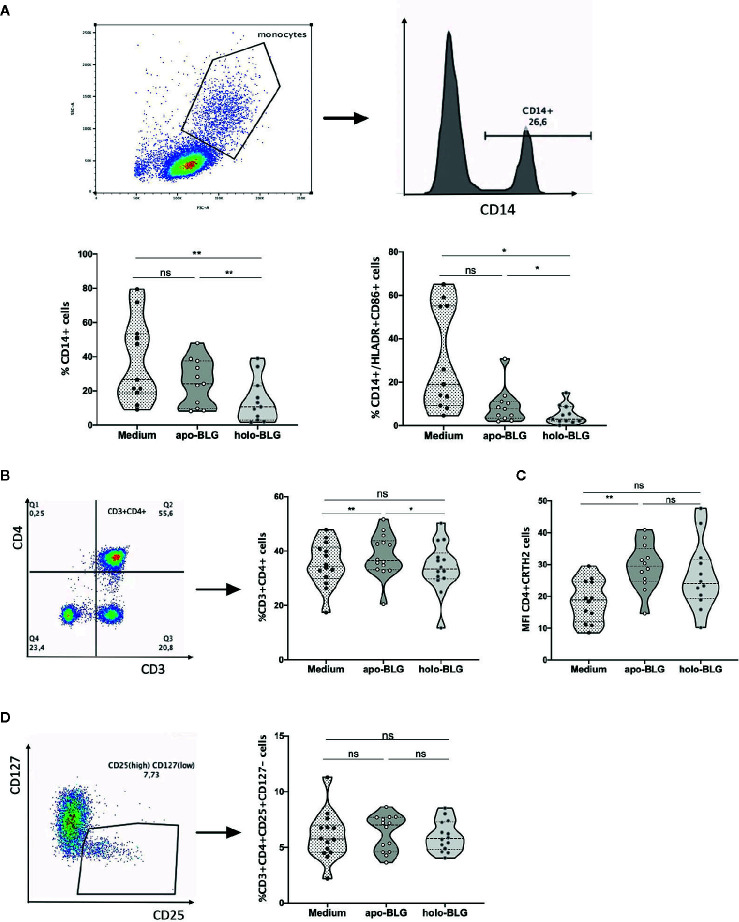
Holo-BLG hinders antigen presentation and decreases the relative number of Th2 cells. PBMCs from 14 pollen allergic donors were stimulated with apo-BLG or holo-BLG and incubated overnight in iron-free media before flow cytometric analysis. **(A)** CD14 positive cells were gated and further analyzed for their HLADR+CD86+ expression. The percentage of different cell subsets was determined in PBMCs after antigen stimulation: **(B)** CD3+CD4+ cells, **(C)** CD4+CRTH2+ cells, **(D)** CD4+CD25+CD127-cells. Data from four independently performed experiments with a total of 14 subjects are shown. Groups were compared by repeated measures one-way ANOVA following the Tukey multiple comparisons test. *P <.05; **P <.01; ns, non-significant.

We investigated the impact of apo-BLG and holo-BLG on surface marker expression of PBMCs from pollen allergic individuals *in vitro* after 18 h incubation. As depicted in **Figure 7A**, holo-BLG reduced the relative numbers of CD14+ monocytes/macrophages. Consequently, the relative numbers of CD14+ cells, expressing the co-stimulatory molecules, HLADR+ and CD86+, were significantly decreased. Holo-BLG reduced CD14+ expression, which is in line with the already described impact on DCs in an *in vivo* murine model in previous studies (23) and link holo-BLG exposure to an overall reduced antigen presentation capacity.

We further analyzed in greater detail the impact of holo-BLG on the T-cell compartment. As previously published, holo-BLG reduced the relative numbers of T helper cells (CD3+CD4+, **Figure 7B**). Within this compartment, holo-BLG suppressed the apo-BLG induced upregulation of CRTH2 on Th2-cells (**Figure 7C**). In contrast, the relative numbers of regulatory T-cells (CD3+CD4+CD25+CD127−) remained stable and were comparable to PBMCs stimulated with apo-BLG (**Figure 7D**).

Upon assessment of cytokine levels in supernatants of PBMCs, stimulation with holo-BLG showed only a trend towards lower levels of IL-4 and IL-13 (**Supplementary Figure 2**), which might reflect that all donors were allergic, and the cytokine pattern of their PBMCs could not be influenced any further in the *ex vivo* treatment.

## Discussion

Exposure to cattle stables and barns, as a consequence of growing up on a farm, have been reported to protect against allergy in humans (28–30). In addition, several studies have shown that consumption of raw, unprocessed, cow’s milk is one of the distinctive farm factors being inversely associated with allergy and asthma (10, 13, 19). The allergy-protective effect of raw cow’s milk is related to the heat-sensitive native whey proteins (10, 15, 31), which are known to have immunomodulatory function (25, 31, 32). However, also other factors in milk, such as microbial components, fatty acids, TGF-beta, IL-10, IgG, microRNA and oligosaccharides have been discussed to contribute to the protective farm effect (33).

In our previous studies, we showed that proper loading of the lipocalin BLG, the major whey compound of milk, with iron–flavonoid complexes can modulate immune-reactivity and induce immune resilience. Its iron-chelating ligands were found to strongly activate the aryl hydrocarbon receptor (AHR) (23), which is expressed by different immune cells (34–36) and considered anti-inflammatory (37). Ligands such as retinoic acid (22, 24) or iron–flavonoid complexes (23) in the calyx of BLG can mask major T-cell linear epitopes, suggesting an increased stability towards endolysosomal enzymes such as cathepsin S and hampering antigen processing (22). The natural innate function of holo-BLG delivering complexed iron particularly to antigen-presenting cells provides an anti-inflammatory signal and further dampens antigen presentation (38, 39).

Indeed, all tested components are present in milk. BLG content in milk ranges from 2 to 5 g/l (corresponding to 100 to 500 µM BLG) (40); the polyphenol content in milk depends on the forage composition and ranges from 3.7 to 35.8 g per liter milk. Quercetin concentration has been measured in milk to be up to 0.68 g/l (this would correspond to up to 2 mM quercetin) (41). Iron concentrations range from 57 µg to 1,500 µg/l (42) (corresponding to roughly 1 to 26 µM Fe). Polyphenolic compounds are highly available in feed plants and constitute part of regular cow diet (41) and polyphenols, *e.g*. quercetin in milk increases after feeding polyphenol-rich diets to lactating animals (43). There are numerous reports showing the iron-binding abilities of BLG (44–47) as the major component in whey (48), leading to improved iron absorption (49–52). On the other hand, milk processing such as pasteurization has been shown to cause aggregation of whey proteins (16), as well as a decrease in copper and iron content in milk. Therefore, there are numerous indirect evidences that at least BLG from milk is indeed loaded with various ligands and that processing can affect its ligand- and iron-binding properties.

These data explain why raw, unprocessed cow’s milk is protective, despite the presence of BLG, which otherwise is best known as the major milk allergen Bos d 5. Our data propose that loading of BLG with ligands possessing anti-inflammatory properties, such as in unprocessed raw milk, maintains it tolerogenic. The likelihood of losing these ligands is particularly increased during industrial milk processing. Additionally, animal welfare may play a role as stressful situations, infections, and supply of forage may influence the ligand loading process.

In the present *in vivo* study, we demonstrate that prophylactic treatment with holo-BLG protects not only against the onset of allergy to this milk protein itself, but the protective impact of holo-BLG is extended to another independent allergen, Bet v 1, in an antigen-unspecific manner. In previous experiments, we could show that iron–quercetin complexes *per se* did not induce a temperature drop or had any impact on serum antibodies and splenocyte cytokine release. Therefore, FeQ2 was no longer included as control group here. Therefore, we cannot fully exclude that FeQ2 could have an allergy preventive effect in the present experimental setup. Holo-BLG prophylaxis targeted antigen presenting cells such as dendritic cells. Levels of the co-stimulatory molecules CD86 were suppressed on dendritic cells (DCs), blocking the co-stimulatory T-cell activation signals and inducing immune tolerance. Several studies showed that CD86 level is upregulated in patients with asthma and allergic diseases (53–55) and is closely associated with Th2 reactions and airway inflammation (56). Importantly, decreasing the expression of the co-stimulatory molecules CD80 and CD86 in DCs has been reported as a potential target for the treatment of allergic diseases (57, 58).

Also, specific antibody production was prevented by prophylactic exposure to holo-BLG *in vivo*. Binding of FeQ2 complex within the calyx of BLG may render BLG more stable towards cathepsin S degradation during antigen processing, resulting in a hampered T-cell-stimulation in an antigen-specific manner (23). Additionally, holo-BLG might have a direct impact on B-cells as antigen presenting cells itself may affect antibody production. Similar to our previous findings, in our mouse model, holo-BLG prevented allergy development, inhibited antigen-specific antibody generation and abrogated Th2 differentiation and Th2 cytokine release to the same allergen. Holo-BLG also prevented allergy development, inhibited antigen-specific antibody generation to the unrelated pollen allergen Bet v 1. This points towards non-antigen specific protection, presumably *via* tolerogenic aryl hydrocarbon receptor pathways and blocking of mast cell degranulation *via* iron transport into mast cells (23).

In line with the *in vivo* data, *in vitro* stimulation of human PBMCs of allergic patients with holo-, but not apo-BLG, lowered the relative numbers of CD14+ monocytes/macrophages, important contributors to the pathogenesis of allergic asthma (59, 60). Consequently, this population revealed less CD86 surface expression, demonstrating an immunosuppressive effect with impaired antigen presentation. Additionally, holo-BLG was able to reduce the relative numbers of CD3+CD4+ T-cells compared to cells stimulated with apo-BLG, and a significant increase of CRTH2 expression was inhibited on CD3+CD4+ T-cells. CRTH2 induces Th2 cells to release type 2 cytokines and is involved in recruiting and activating eosinophils and basophils, which further contribute to amplification of type 2 inflammation (61–64), whereas blocking of CRTH2 by antagonists suppresses allergic inflammation (65, 66). Furthermore, we show that spiked holo-BLG repressed CRTH2 expression and thus seems able to attenuate a Th2-associated response. Stimulation of these PBMCs with holo-BLG showed only a trend towards lower levels of IL-4 and IL-13, which might reflect that all donors were allergic, and the cytokine pattern of their PBMCs could not be influenced any further by *ex vivo* treatment.

Intriguingly, when the major milk protein BLG in its holo- form transports ligands to immune cells, this will result in innate immune resilience (23, 24). In line with this novel molecular concept, the results of our current *in vivo* study suggest that exposure to the loaded holo-BLG can in an antigen-nonspecific manner protect against allergic sensitization. This phenomenon might be due to targeted delivery of ligands from BLG to immune cells, supplementation of intracellular iron, and quercetin activating the AhR, altogether synergizing in non-antigen-specific immune tolerance.

The results of the present study propose that ligand-bound BLG contributes to the protective farm effect, as it is a major constituent of raw milk, and its abundant presence has been identified in the dust of cattle farms (26) and (Pali-Scholl et al., manuscript in review).

Further studies need to address i) how farming conditions, the cows’ health status, as well as the forage composition, affect the richness of ligands available for BLG in milk, and ii) how industrial processing can be adapted to prevent changes in the ligand composition and protein integrity.

## Data Availability Statement

The raw data supporting the conclusions of this article will be made available by the authors, without undue reservation.

## Ethics Statement

The studies involving human participants were reviewed and approved by the institutional ethics committee of the Medical University of Vienna and conducted in accordance with the Helsinki Declaration of 1975. The patients/participants provided their written informed consent to participate in this study. The animal study was reviewed and approved by the Animal Experimentation Ethics Committee of the University of Vienna and the Ministry of Education, Science and Culture (BMWF-66.009/0133-WF/V/3b/2016).

## Author Contributions

SMA conducted all mouse and cell stimulation experiments, performed statistical analysis, provided support and wrote the manuscript. IP-S obtained the legal requirements for and helped in the mouse experiments, provided support and contributed to writing and editing of the manuscript. KH contributed in the mouse experiments and to writing. GH provided support and contributed to the writing. ME-B provided support and contributed to writing. FR-W contributed in the mouse experiments, conceived, and directed the research, interpreted the data and contributed in writing. EJ-J financed and directed research, designed the experiments, and contributed in manuscript writing. All authors contributed to the article and approved the submitted version.

## Funding

The work was supported by the Austrian Science Fund FWF, SFB F4606-B28, by Biomedical International R+D GmbH, Vienna, Austria, and by Bencard Allergie GmbH, Munich, Germany. SMA was also supported by a grant from the Ministry of Higher Education, Egypt.

## Conflict of Interest

EJ-J is a shareholder of Biomedical International R+D GmbH, Vienna, Austria.

EJ-J and FR-W are inventors of EP2894478, LCN2 as a tool for allergy diagnostic and therapy. EP 14150965.3, Year: 01/2014; US 14/204,570, owned by Biomedical International R+D GmbH, Vienna, Austria.

The authors declare that this study received funding from Biomedical International R+D GmbH, Vienna, Austria and Bencard Allergy GmbH, Germany. Both funders provided research grants for the laboratory work and employment of S.M.A. as PhD student.

The remaining authors declare that the research was conducted in the absence of any commercial or financial relationships that could be construed as a potential conflict of interest.

## Supplementary Material

Supplementary Figure 1 Holo-BLG treatment protected against acute allergic symptoms in an antigen-unspecific manner. Pretreated mice were sensitized to Betv1, thereafter challenged with Bet v 1 and anaphylactic shock symptom score (Kruskal–Wallis test) was monitored in a blinded fashion. Representative data from two independent experiments are shown. Medians with interquartile ranges are represented in box whisker plots; **P* < 0.05; ns = non-significant.

Supplementary Figure 2 PBMCs from 14 pollen allergic donors were stimulated with apo-BLG or holo-BLG and incubated overnight in iron-free media. Supernatants of PBMCs were assessed for **(A)** IL-2, **(B)** IL-6, **(C)** IL-4, **(D)** IL-13, **(E)** IL-10 and **(F)** IFN-*γ* by multiplexing in FACS. Data from four independently performed experiments with a total of 14 subjects are shown. Groups were compared by repeated measures one-way ANOVA following the Tukey multiple comparisons test. *P <.05; **P <.01; ****P < 0.0001; ns = non-significant.
