# Supplementary figures and images for: Bovine Holo-Beta-Lactoglobulin Cross-Protects Against Pollen Allergies in an Innate Manner in BALB/c Mice: Potential Model for the Farm Effect
Source: Front Immunol. 2021 Mar 5;12:611474. doi: 10.3389/fimmu.2021.611474 (PMC7977286; doi:10.3389/fimmu.2021.611474)

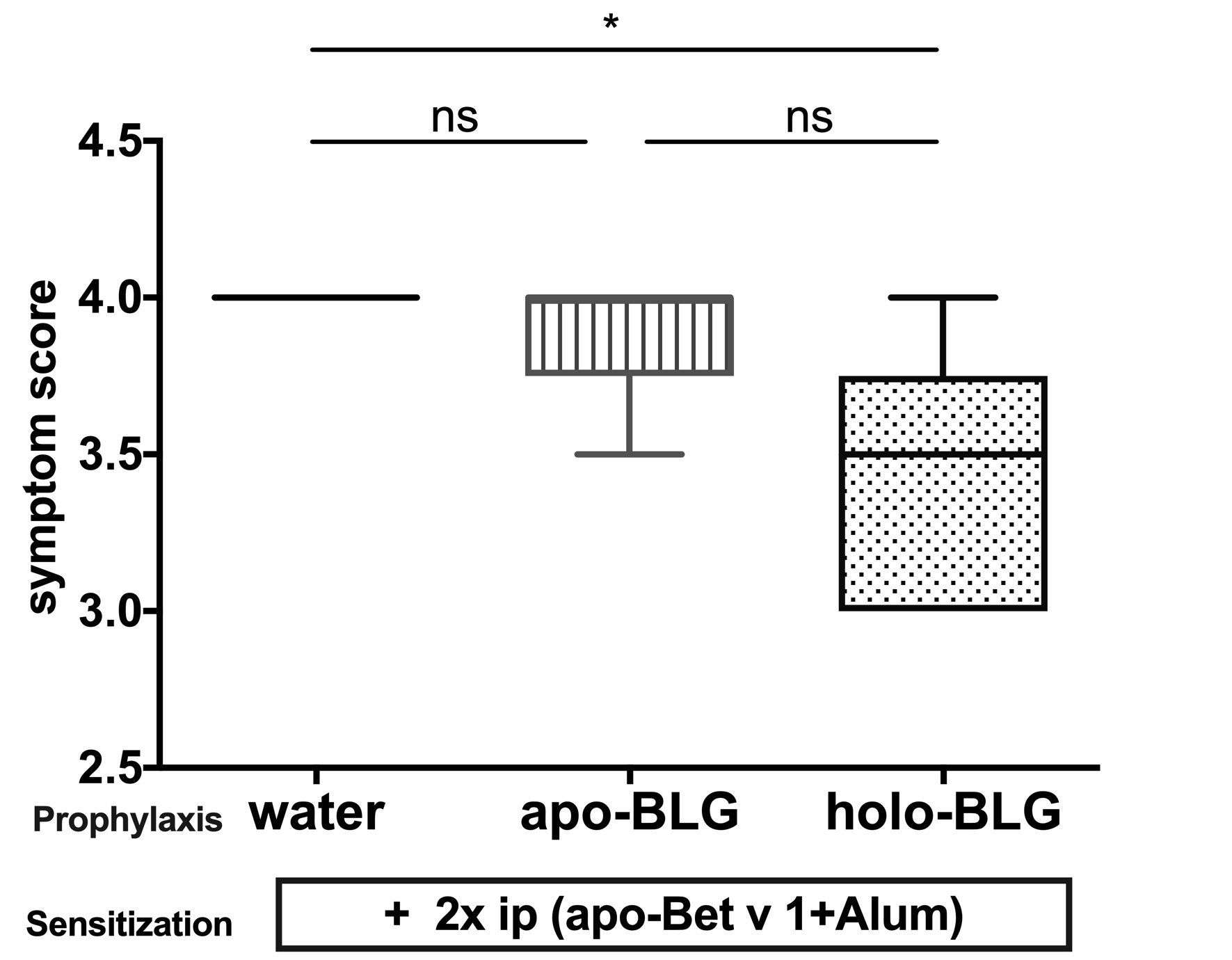

Supplement: Supplementary Figure 1 — Holo-BLG treatment protected against acute allergic symptoms in an antigen-unspecific manner. Pretreated mice were sensitized to Betv1, thereafter challenged with Bet v 1 and anaphylactic shock symptom score (Kruskal–Wallis test) was monitored in a blinded fashion. Representative data from two independent experiments are shown. Medians with interquartile ranges are represented in box whisker plots; *P < 0.05; ns = non-significant. [file Image_1.jpeg]

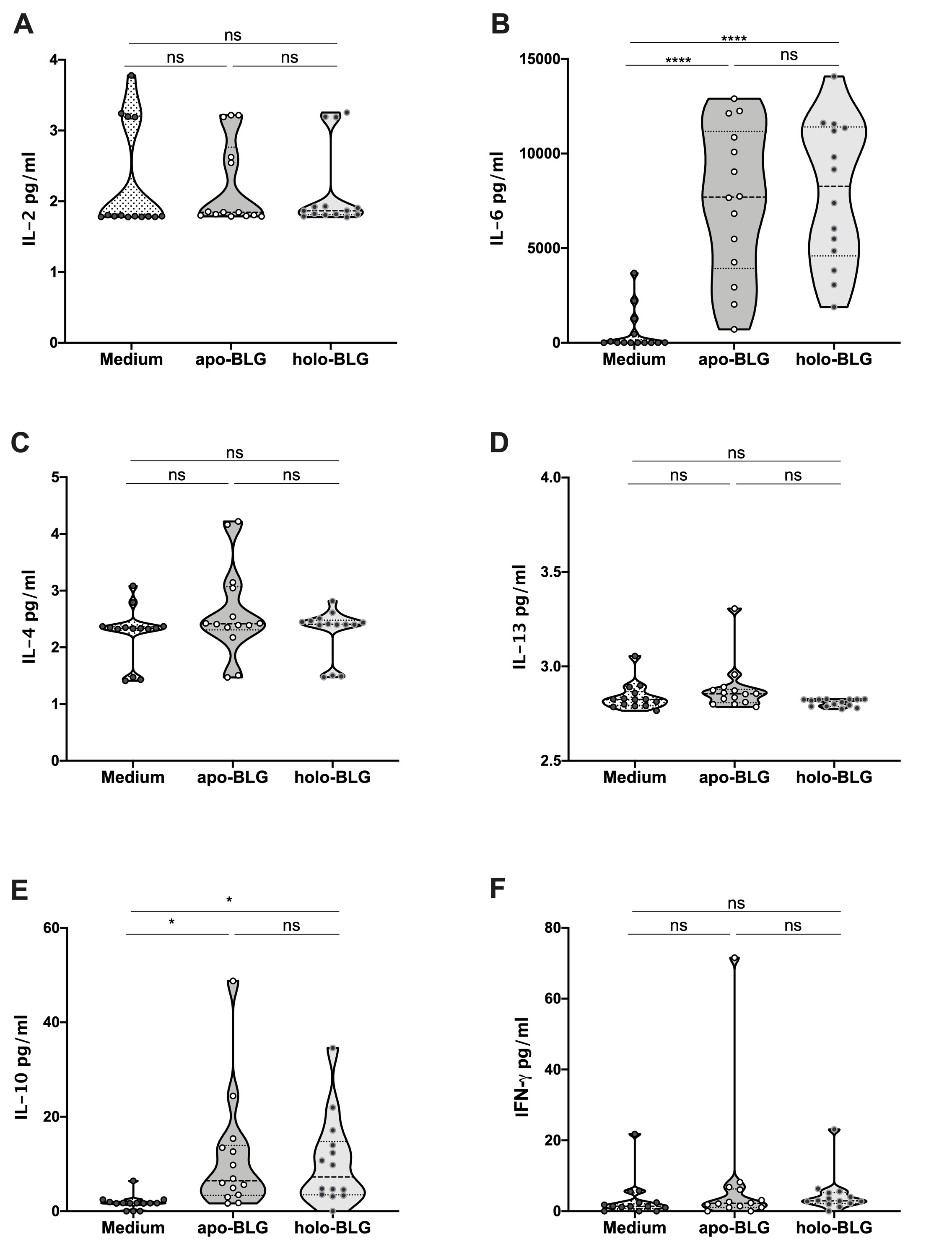

Supplement: Supplementary Figure 2 — PBMCs from 14 pollen allergic donors were stimulated with apo-BLG or holo-BLG and incubated overnight in iron-free media. Supernatants of PBMCs were assessed for (A) IL-2, (B) IL-6, (C) IL-4, (D) IL-13, (E) IL-10 and (F) IFN-γ by multiplexing in FACS. Data from four independently performed experiments with a total of 14 subjects are shown. Groups were compared by repeated measures one-way ANOVA following the Tukey multiple comparisons test. *P <.05; **P <.01; ****P < 0.0001; ns = non-significant. [file Image_2.jpeg]
